# High-Efficiency Capture of Drug Resistant-Influenza Virus by Live Imaging of Sialidase Activity

**DOI:** 10.1371/journal.pone.0156400

**Published:** 2016-05-27

**Authors:** Yuuki Kurebayashi, Tadanobu Takahashi, Chihiro Tamoto, Keiji Sahara, Tadamune Otsubo, Tatsuya Yokozawa, Nona Shibahara, Hirohisa Wada, Akira Minami, Kiyoshi Ikeda, Takashi Suzuki

**Affiliations:** 1 Department of Biochemistry, School of Pharmaceutical Sciences, University of Shizuoka, Shizuoka-shi, Shizuoka, Japan; 2 Shizuoka Institute of Environment and Hygiene, Shizuoka-shi, Shizuoka, Japan; 3 Department of Organic Chemistry, School of Pharmaceutical Sciences, Hiroshima International University, Kure-shi, Hiroshima, Japan; 4 Shizuoka City Institute of Environmental Sciences and Public Health, Shizuoka-shi, Shizuoka, Japan; University of Rochester Medical Center, UNITED STATES

## Abstract

Influenza A and B viruses possess a neuraminidase protein that shows sialidase activity. Influenza virus-specific neuraminidase inhibitors (NAIs) are commonly used for clinical treatment of influenza. However, some influenza A and B viruses that are resistant to NAIs have emerged in nature. NAI-resistant viruses have been monitored in public hygiene surveys and the mechanism underlying the resistance has been studied. Here, we describe a new assay for selective detection and isolation of an NAI-resistant virus in a speedy and easy manner by live fluorescence imaging of viral sialidase activity, which we previously developed, in order to achieve high-efficiency capture of an NAI-resistant virus. An NAI-resistant virus maintains sialidase activity even at a concentration of NAI that leads to complete deactivation of the virus. Infected cells and focuses (infected cell populations) of an oseltamivir-resistant virus were selectively visualized by live fluorescence sialidase imaging in the presence of oseltamivir, resulting in high-efficiency isolation of the resistant viruses. The use of a combination of other NAIs (zanamivir, peramivir, and laninamivir) in the imaging showed that the oseltamivir-resistant virus isolated in 2008 was sensitive to zanamivir and laninamivir but resistant to peramivir. Fluorescence imaging in the presence of zanamivir also succeeded in selective live-cell visualization of cells that expressed zanamivir-resistant NA. Fluorescence imaging of NAI-resistant sialidase activity will be a powerful method for study of the NAI resistance mechanism, for public monitoring of NAI-resistant viruses, and for development of a new NAI that shows an effect on various NAI-resistant mutations.

## Introduction

Influenza viruses circulate worldwide and can affect every year up to 10% of the world’s population [1]. An influenza epidemic often causes economic impact and public health problem. Antiviral drugs for influenza are available in some countries and may reduce severe complications and deaths. But some influenza viruses can develop resistance to the antiviral medicines, limiting the effectiveness of treatment [2].

Neuraminidase (NA) is a major surface glycoprotein of the influenza A and B viruses and expresses on the surface of the virus-infected cell at a high amount. NA shows sialidase activity that hydrolyzes the bond between galactose and terminal sialic acid in the glycochain. The sialidase activity of NA is critical for the virus replication cycle and therefore has been considered a suitable target for designing agents against influenza viruses. NA inhibitors (NAIs), which are sialic acid analogues and specific competitive enzymatic inhibitors against influenza virus NA, are clinically used for prevention and treatment of influenza [3–5]. Several countries possess stockpiles of NAIs because of their effectiveness and for preparation in prevention of and treatment of patients in an influenza pandemic [6–9]. However, NA genes sometimes acquire resistance mutations against NAIs [10, 11]. Resistance against NAIs, such as oseltamivir and zanamivir, had not been thought to be an important problem in clinical treatment because the resistance mutations reduce sialidase activity of NA to some extent [12–16]. In mouse and ferret infection models of oseltamivir-resistant viruses, the NAI-resistant viruses had also been reported to be unfit for efficient virus replication and to be poorly transmissible [13, 15]. However, an oseltamivir-resistant H1N1 virus emerged in North Europe in 2007 and then spread worldwide in the 2008–2009 season [17, 18]. In a seasonal H1N1 influenza A virus, an amino acid alteration of histidine to tyrosine at position 275 (H275Y, based on N1NA amino acid numbering) in the N1NA was critical for oseltamivir resistance [3, 9–20]. Additional amino acid alterations (R222Q and V234M), which increased both sialidase activity and surface expression of NA, conferred ability of efficient virus replication, allowing worldwide spread of the oseltamivir-resistant H1N1 virus [21]. Immediately after a pandemic occurrence of the oseltamivir-sensitive H1N1 virus (pdm09) transmitted from pigs in 2009, epidemic oseltamivir-resistant H1N1 virus disappeared [22]. Epidemic H3N2 virus and influenza B virus also showed oseltamivir resistance and/or zanamivir resistance, although their frequencies are low. [23, 24]. Thus, in nature, an NAI-resistant virus occurs in epidemics. A large epidemic or pandemic of an NAI-resistant virus might have serious effects on societies and economies worldwide. A national project for the establishment of an applicable NAI stockpile and appropriate usage of NAIs in clinical treatment need reliable and up-to-date epidemiological information. Establishment of highly efficient methods for detection and isolation of NAI-resistant viruses will greatly contribute to rapid and large-scale collection of epidemiological NAI-resistance information and to studies aimed at elucidation of the NAI resistance mechanism.

Methods for detection of NAI-resistant viruses can be divided into enzymatic assays and genotypic assays of viral NA [10, 12, 20, 22]. An enzymatic assay is used to measure the degrees of inhibition of sialidase activity in the presence of NAIs. By using an enzymatic assay, the 50% inhibition concentration (IC_50_) value of each NAI can be determined and susceptibilities to NAIs can be compared. However, an enzymatic assay involves troublesome procedures for hygiene workers who must examine numerous samples, such as procedures for measurement of sialidase activity and for growth and isolation of a virus. A genotypic assay is used to detect a mutation of the NA gene that confers NAI resistance. Most genotypic assays are used for detection of H275Y conferring oseltamivir resistance. Genotypic assays are generally used in laboratories of environmental hygiene including regional laboratories because they easily enable highly sensitive detection of an NAI-resistance mutation directly from clinical samples or from virus cultures by genetic analysis and/or real-time PCR of the NA gene. However, the application of a genotypic assay is limited to already known mutations and cannot be used to evaluate NAI resistance attributed to unknown mutations. In addition, a genotypic method cannot be used to compare NAI susceptibilities such as IC_50_ values. In the case of a mixture of NAI-sensitive and NAI-resistant viruses, each virus strain must be isolated by the conventional plaque-forming assay before the enzymatic assay or genotypic assay [25–28]. Thus, it is necessary to establish a novel enzymatic assay for highly efficient detection and isolation of NAI-resistant viruses that may possess unknown resistance mutations.

We previously developed a sialidase fluorescence imaging reagent, 2-(benzothiazol-2-yl)-4-bromophenyl-5-acetamido-3,5-dideoxy-α-d-*glycero*-d-*galacto*-2-nonulopyranosidonic acid (BTP3-Neu5Ac) [29, 30]. BTP3-Neu5Ac is a water-soluble non-fluorescent compound. After removal of α-d-*N*-acetylneuraminic acid (Neu5Ac) by sialidases from viruses including influenza A and B viruses, the product BTP3 is a benzothiazolylphenol-based water-insoluble fluorescent compound [31]. BTP3 locally deposits on existing sites of sialidase activity of NA expressed on the influenza virus-infected cells, resulting in sensitive, rapid and easy fluorescence imaging of live influenza virus-infected cells [29, 32–34]. Fluorescence imaging of the infected cells by BTP3-Neu5Ac is inhibited by adding influenza virus-specific NAI. Therefore, it is predicted that BTP3-Neu5Ac can utilize for selective detection of NAI-resistant virus-infected cells because of maintenance of its sialidase activity even in the presence of an NAI, and that it enables selective live fluorescence imaging of plaques (focuses) formed by NAI-resistant viruses in a conventional plaque-forming assay. Live-focus fluorescence imaging is also applicable to high-efficiency isolation of NAI-resistant viruses. This paper exhibits the usefulness of BTP3-Neu5Ac for selective detection and isolation of an NAI-resistant influenza virus by live-cell or live-focus fluorescence imaging.

## Materials and Methods

### Cells, viruses, and NAIs

Cells were obtained from ATCC. Madin-Darby canine kidney (MDCK) cells (ATCC-CCL-34) were grown in a minimum essential medium (MEM) supplemented with 5%fetal bovine serum (FBS). African green monkey kidney COS7 cells (ATCC-CRL-1651, one of the SV40-transfected African green monkey kidney fibroblast-like CV-1 cell lines) were grown in a Dulbecco's Modified Eagle Medium (DMEM) supplemented with 10% FBS. Influenza A virus strains, oseltamivir-sensitive A/Shizuoka/838/2009 (H1N1pdm) (838) and oseltamivir-resistant A/Shizuoka/738/2008 (H1N1) (738), were grown in MDCK cells. Hemagglutination units (HAU) of the viruses were determined as described previously [35]. HAU were expressed as the highest dilution of the virus suspension giving complete agglutination of guinea-pig erythrocytes. Oseltamivir (carboxylate), zanamivir, peramivir, and laninamivir were purchased from Chemscene LLC, LKT Laboratories Inc., AdooQ BioScience, and Toronto Research Chemicals Inc., respectively. Oseltamivir carboxylate was used in all experiments as an active form showing sialidase inhibitory activity.

### Plaque-forming assay

Virus titers were determined by a plaque-forming assay [36]. Viruses were ten-fold serially diluted in a serum-free medium (SFM), Hybridoma-SFM (Invitrogen Corp., CA, USA). A monolayer of MDCK cells on a 6-well plate was infected with 1 ml of the virus dilutions at 37°C for 30 min. After washing with phosphate buffered saline (PBS), the cells were cultured in 4 ml of an SFM containing 0.5% agarose and 2 μg/ml acetylated trypsin at 37°C for an additional 3 days. Plaque-forming units (pfu) were calculated as virus titers from the number of viral plaques.

### Sialidase inhibition assay

Viruses were standardized to equivalent NA enzyme activity in the linear range of the curve. Forty microliters of virus suspension in PBS was mixed with 5 μl of ten-fold dilutions of oseltamivir or distilled water only on a 96-well black plate (BD FALCON, NJ, USA). The mixture was incubated at 37°C for 15 min. Five microliters of 1 mM 2'-(4-methylumbelliferyl)-α-d-*N*-acetylneuraminic acid (4MU-Neu5Ac) or 1 mM BTP3-Neu5Ac was added on ice and then incubated at 37°C for 1 hr. Enzyme reaction for 4MU-Neu5Ac was stopped by 50 μl of 100 mM sodium carbonate buffer (pH 10.7). Fluorescence from sialidase reaction was quantified by using a microplate reader (Infinite 200 multi-mode Reader, Tecan, Männedorf, Switzerland) with wavelengths of excitation and emission at 355/460 nm for 4-metylumbelliferone (4MU) and at 372/526 nm for BTP3. The concentration of NAI that reduced NA enzyme activity by 50% relative to a control mixture with no NAI was determined as IC_50_ by plotting the percent inhibition of the activity at respective NAI concentrations using GraphPad Prism 5 software (GraphPad Software, La Jolla, CA).

### Live-cell fluorescence imaging of oseltamivir-resistant virus-infected cells

MDCK cells on a 48-well plate (5 × 10^4^ cells/well) were cultured for 24 hr. The cells were infected with oseltamivir-resistant 738 or -sensitive 838 at a multiplicity of infection (MOI) of 0.01 in 250 μl of an SFM and incubated at 37°C for 12 hr. After removing the supernatant, the infected cells were incubated with 150 μl of 20 μM BTP3-Neu5Ac with or without oseltamivir or zanamivir (each 100 nM for final concentrations) in an SFM at 37°C for 10 min. Fluorescent images of the cells were observed under UV irradiation by using an IX71 fluorescence microscope (Olympus Co., Ltd., Tokyo, Japan) equipped with fluorescent filters (U-MWU2, DM400, BP330-385, and BA420).

### Live-cell fluorescence imaging of cells transfected with NAI-resistant NA genes

Site-specific mutations of NA genes were introduced as described previously [37]. The mutated NA genes in pGEM-T easy vector (Promega, Madison, WI, USA) were amplified and inserted into the multi cloning site of pCAGGS/MCS vector between *Eco*R I and *Xho* I. COS7 cells at 80% confluency on a 48-well plate were transfected with 500 ng/well of each pCAGGS expression vector using TransIT-LT1 (Mirus, Madison, WI, USA) according to the manufacturer’s instructions and incubated for 24 hr at 37°C. After removing the supernatant, the infected cells were incubated with or without 100 nM oseltamivir or 100 nM zanamivir in an SFM at 37°C for 5 min. Then, BTP3-Neu5Ac (20 μM for final concentration) was added to cell culture medium at 37°C for 5 min. Fluorescent images of the cells were observed under UV irradiation by using an IX71 fluorescence microscope equipped with fluorescent filters (U-MWU2, DM400, BP330-385, and BA420).

### Live-focus fluorescence imaging of oseltamivir-resistant virus

A monolayer of MDCK cells on a 12-well plate was infected with oseltamivir-resistant 738 or -sensitive 838 at 20 pfu/well in 500 μl of an SFM at 37°C for 30 min and cultured in 2 ml of an SFM containing 0.5% agarose and 2 μg/ml acetylated trypsin. After 2 days at 37°C, 50 μl of 2 mM BTP3-Neu5Ac with each NAI (10, 100, and 1000 nM for final concentrations), oseltamivir, zanamivir, peramivir, and laninamivir, was added onto the overlaid agarose-containing SFM. After 1 hr at 37°C, fluorescent images of the plate were observed on a UV Transilluminator (UV BOX-W, NATURAL IMMUNITY, Tokyo, Japan) at 365 nm.

### Selective detection of oseltamivir-resistant virus-formed focuses using live-focus fluorescence imaging

Mixtures of oseltamivir-resistant 738 and -sensitive 838 were prepared at three ratios of 100:0, 20:80, and 4:96 in virus infectious titers, respectively. A monolayer of MDCK cells in a 6-well plate was infected with each mixture (200 pfu/well) at two wells, respectively. Cells were infected at 37°C for 30 min and cultured in 4 ml of an SFM containing 0.5% agarose and 2 μg/ml acetylated trypsin. After 2 days at 37°C, 100 μl of 2 mM BTP3-Neu5Ac with or without oseltamivir (1000 nM for a final concentration) was added onto the overlaid agarose-containing SFM. After 1 hr at 37°C, fluorescent images of the plate were observed on a UV Transilluminator at 365 nm.

### Selective isolation of oseltamivir-resistant viruses using live-focus fluorescence imaging

A mixture of oseltamivir-resistant 738 and -sensitive 838 was prepared at a ratio of 1:1 in virus infectious titers. A monolayer of MDCK cells in a 6-well plate was infected with the mixture (40 pfu/well). Cells were infected at 37°C for 30 min and cultured in 4 ml of an SFM containing 0.8% agarose and 2 μg/ml acetylated trypsin. After 2 days at 37°C, 100 μl of 2 mM BTP3-Neu5Ac with or without oseltamivir (1000 nM for a final concentration) was added onto the overlaid agarose-containing SFM. After 1 hr at 37°C, fluorescent images of the plate were observed on a UV Transilluminator at 365 nm.

Viruses were isolated from eight fluorescent focuses in live-focus fluorescence imaging in the presence of oseltamivir. The isolates were grown in MDCK cells within 3 passages until RNA extraction. The experiment was repeated three times (isolation from total 24 fluorescent focuses).

### Genetic detection of oseltamivir-resistant virus

Viral RNA was extracted from virus cultures by using RNeasy mini Kit (Qiagen, CA, USA) according to the manufacturer’s instructions. To distinguish genes of oseltamivir-resistant 738 and -sensitive 838, oseltamivir resistance mutation of H275Y (based on N1NA amino acid numbering) in NA was detected by one-step RT-PCR using PrimeScript^™^ One Step RT-PCR Kit Ver. 2 (TaKaRa Bio, Shiga, Japan) with the following primer pairs: 08seasonNA-275Y-F (5’-GAGTTGAATGCACCCAATTTTTAT-3’) and UniNA-R (5’-TATTGGTCTCAGGGAGCAAAAGCAGGAGT-3’) for 275Y detection of 738, and 09pdmNA-275H-F (5’-AAATGAATGCCCCTAATTATCAC-3’) and UniNA-R for 275H detection of 838. RT-PCR were performed by using a TP600 PCR machine (TaKaRa Bio, Shiga, Japan) with 1 cycle at 50°C for 30 min and 1 cycle at 94°C for 2 min (RT), 28 cycles at 94°C for 30 sec, 58°C for 30 sec, and 72°C for 45 sec (PCR), and 1 cycle at 72°C for 45 sec. The PCR products were analyzed by 1% agarose gel electrophoresis.

## Results

### IC_50_ values of oseltamivir-resistant and -sensitive viruses using BTP3-Neu5Ac

**Fig 1:**
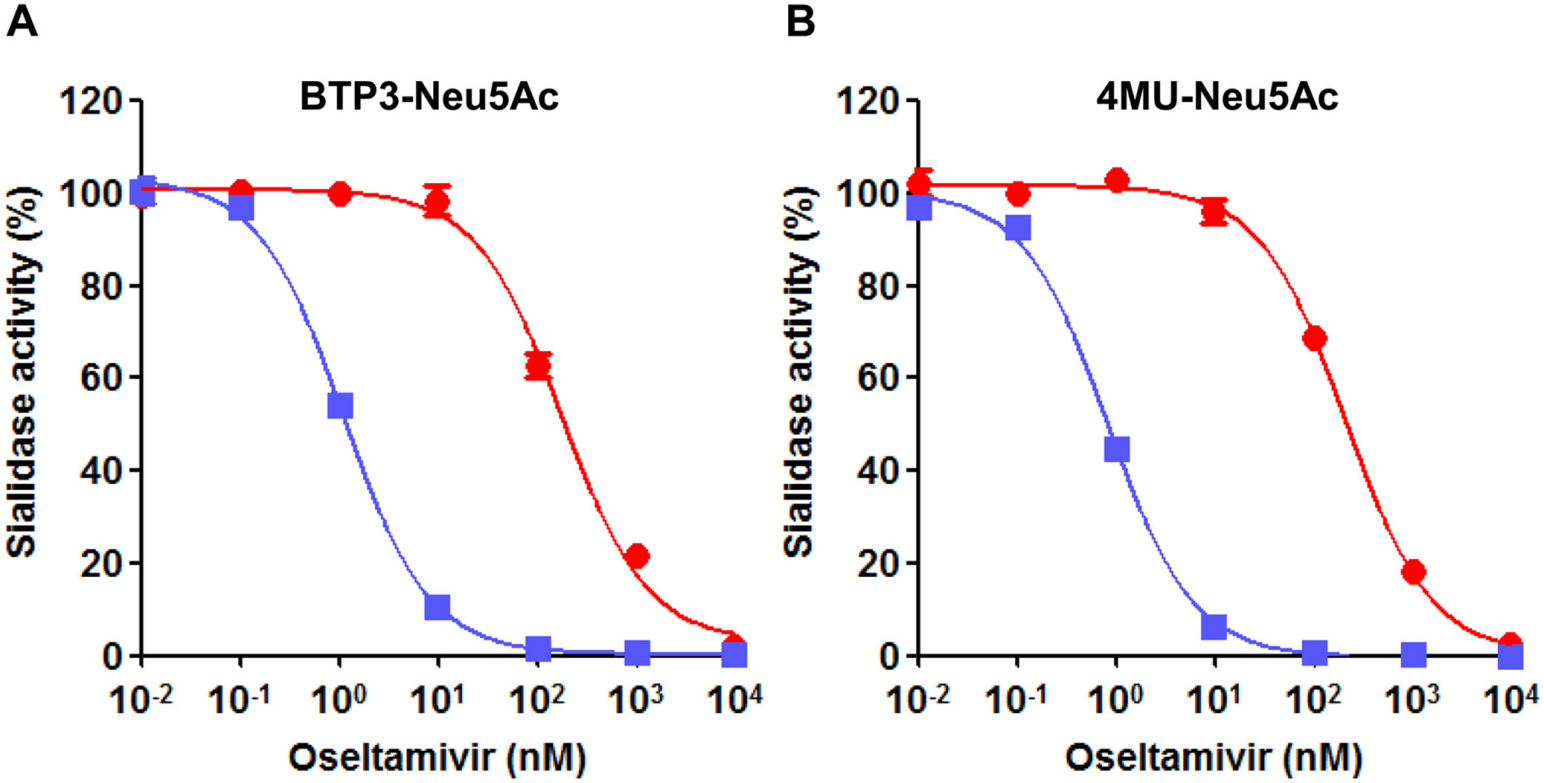
Inhibition effect of oseltamivir for sialidase activities of oseltamivir-resistant and -sensitive viruses using 4MU-Neu5Ac or BTP3-Neu5Ac. Sialidase activities of oseltamivir-resistant 738 (red circle) and -sensitive 838 (blue square) were measured in the presence of ten-fold serial dilutions of oseltamivir by using BTP3-Neu5Ac (A) or 4MU-Neu5Ac (B). Sialidase activity (%) was expressed as a percentage of the activity in the absence of oseltamivir. Each value is the mean and standard deviation from triplicate measurements.

An influenza A virus- and B virus-specific NAI, oseltamivir (carboxylate form showing sialidase inhibitory activity), was used in this study for detection for oseltamivir-resistant influenza A virus A/Shizuoka/738/2008 (H1N1) (abbreviated as 738), which was an epidemic virus in nature during the 2008–2009 season in Japan. We have reported that zanamivir, one of influenza A virus- and B virus-specific NAIs, inhibited live-cell fluorescence imaging of influenza A virus-infected cells for BTP3-Neu5Ac usage [29]. However, we do not confirm inhibition of the fluorescence visualization by oseltamivir for BTP3-Neu5Ac usage. Therefore, we firstly measured IC_50_ values of oseltamivir using BTP3-Neu5Ac. IC_50_ values (means ± standard deviations) of oseltamivir for oseltamivir-resistant 738 and -sensitive A/Shizuoka/838/2009 (H1N1pdm09) (abbreviated as 838) were 175.7 nM (95% CI, 145.9–211.5) and 1.122 (95% CI, 1.021–1.232), respectively (Fig 1A). We also measured IC_50_ values using 4MU-Neu5Ac, which has been commonly used to measure IC_50_ values of NAIs. IC_50_ values of oseltamivir for 738 and 838 were 209.2 nM (95% CI, 183.0–239.2) and 0.8393 (95% CI, 0.7503–0.9387), respectively (Fig 1B). IC_50_ values of oseltamivir for BTP3-Neu5Ac usage were similar to those for 4MU-Neu5Ac usage. IC_50_ values for oseltamivir-resistant 738 were more than 100-fold higher compared to those on oseltamivir-sensitive 838. The results shown in Fig 1 suggested that 100 nM oseltamivir (final concentration) was an effective concentration for selective detection of oseltamivir resistance, because viral sialidase activity at that concentration was completely inhibited in 838 and significantly maintained in 738.

### Selective live-cell fluorescence imaging of oseltamivir-resistant virus-infected cells

**Fig 2:**
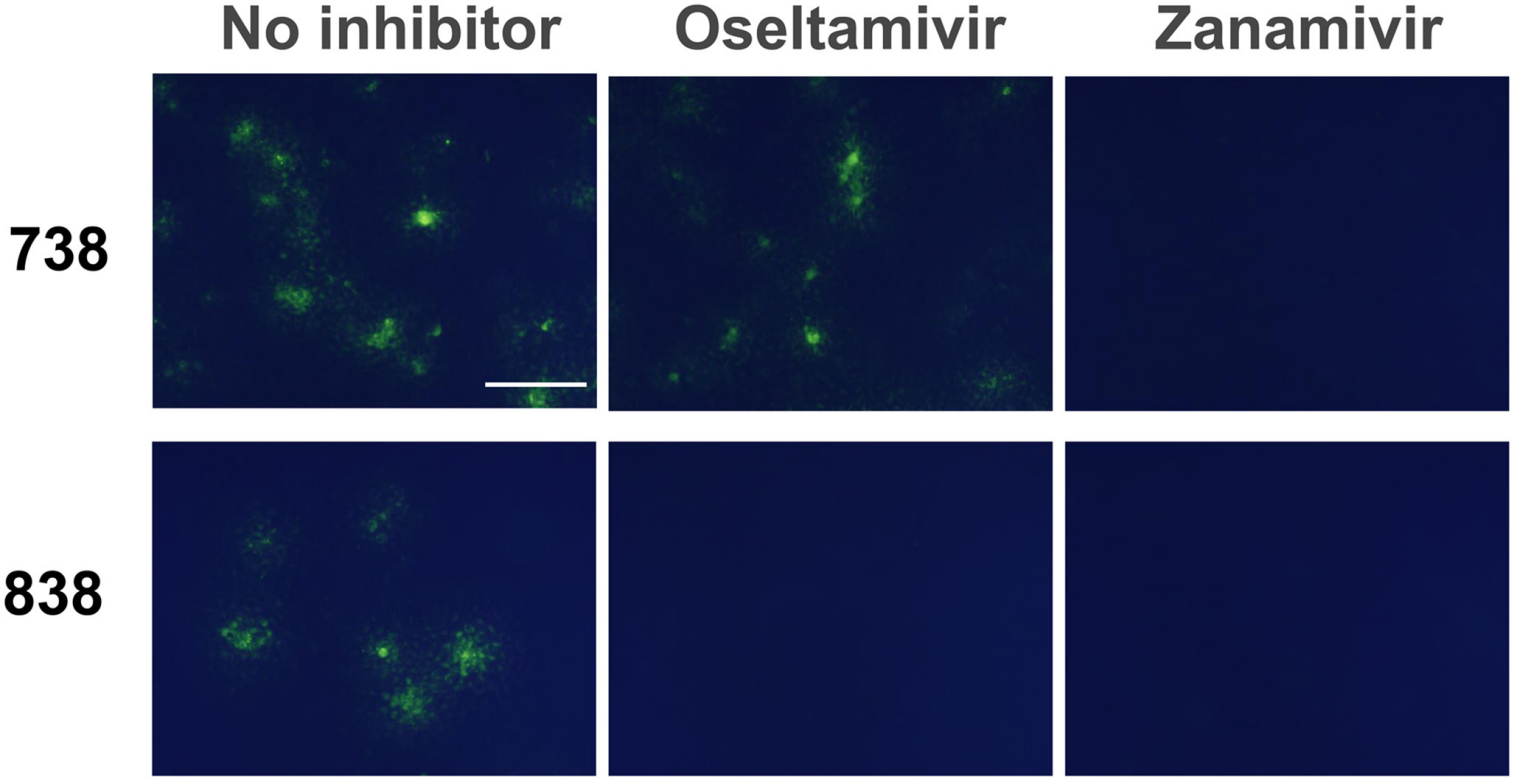
Live-cell fluorescence imaging of infected cells of oseltamivir-resistant virus. MDCK cells were infected with oseltamivir-resistant 738 and -sensitive 838. After incubation at 37°C for 12 hr, the cells were incubated with BTP3-Neu5Ac with or without oseltamivir or zanamivir (each 100 nM for final concentrations) in an SFM at 37°C for 10 min. Fluorescent images of the cells were observed under UV irradiation by using a fluorescence microscope. A scale bar indicates 200 μm.

We examined selective fluorescent visualization of oseltamivir-resistant virus-infected cells using BTP3-Neu5Ac with oseltamivir. MDCK cells were infected with oseltamivir-resistant 738 or -sensitive 838. The infected cells at 12 hr postinfection were incubated with BTP3-Neu5Ac with or without oseltamivir or zanamivir (each 100 nM for final concentrations) at 37°C for 10 min (Fig 2). Oseltamivir-resistant 738-infected cells were fluorescent-visualized even in the presence of oseltamivir, but oseltamivir-sensitive 838-infected cells were not. Nearly equal amounts of virus infections could be confirmed by fluorescence in both infected cells of 738 and 838 in the absence of NAIs. Complete inhibition of fluorescent visualization in the presence of zanamivir indicates that the fluorescence is dependent on viral sialidase activity of the infected cells. As a result, we succeeded in selective live-cell fluorescence imaging of oseltamivir-resistant virus-infected cells.

**Fig 3:**
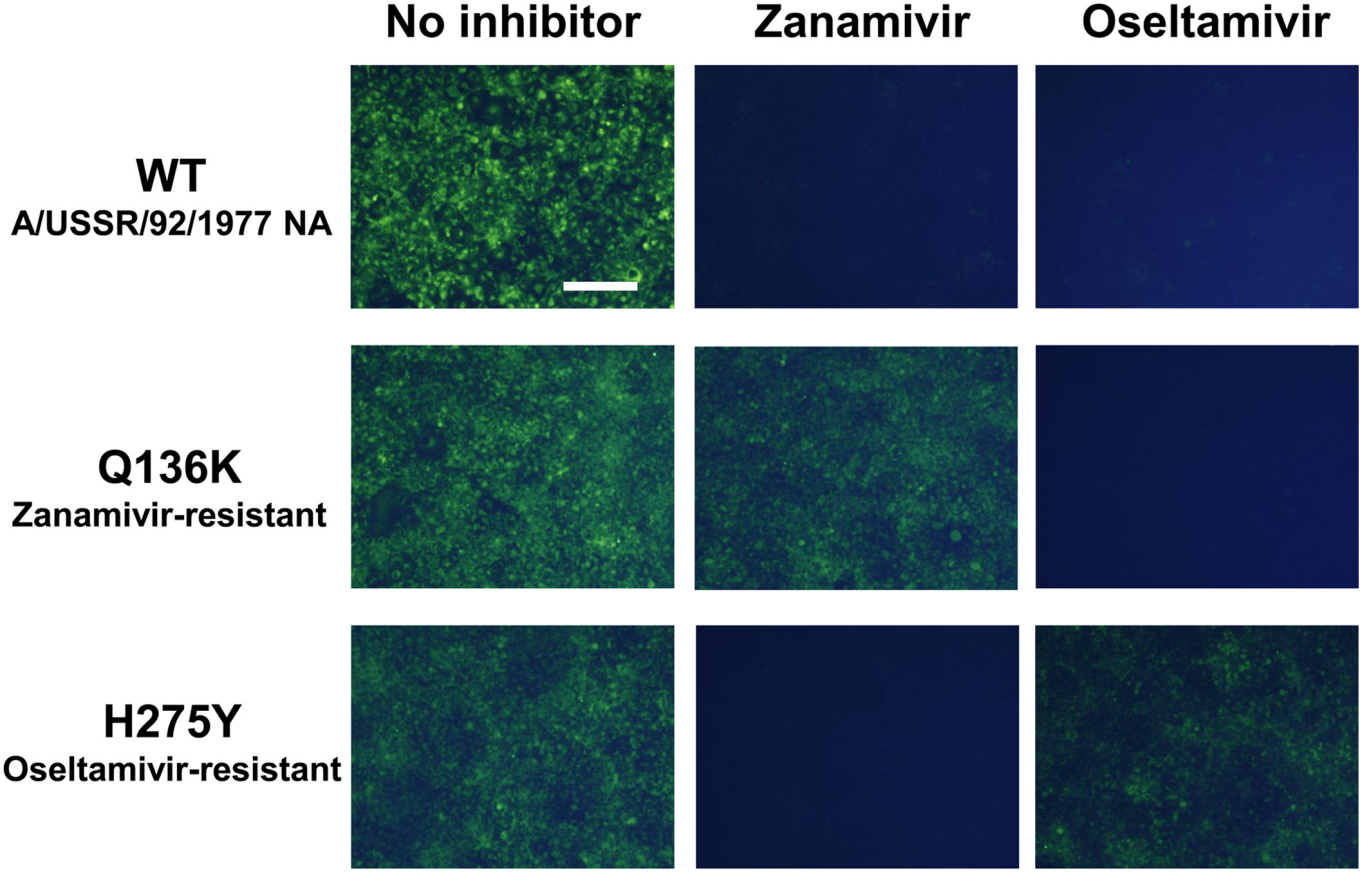
Live-cell fluorescence imaging of cells transfected with NAI-resistant NA genes. Zanamivir-resistant mutation (Q136K) or oseltamivir-resistant mutation (H275Y) were introduced in NA gene of zanamivir and oseltamivir-sensitive virus A/USSR/92/1977 (H1N1). The NA genes were transfected to COS7 cells. After 24 hr, the NA-expressed cells were incubated with 100 nM zanamivir or oseltamivir in an SFM at 37°C for 5 min. Then, BTP3-Neu5Ac (20 μM for final concentration) was added to cell culture medium at 37°C for 5 min. Fluorescent images of the cells were observed under UV irradiation by using a fluorescence microscope. A scale bar indicates 200 μm.

To examine whether the live-cell fluorescence imaging can be applicable to zanamivir resistance, we gave NA of zanamivir-sensitive virus A/USSR/92/1977 (H1N1) the property of zanamivir resistance by amino acid substitution of Q136K [11, 12]. BTP3-Neu5Ac treatment in the presence of zanamivir succeeded selective live-cell fluorescence imaging of cells transfected with zanamivir-resistant NA gene. We also confirmed that BTP3-Neu5Ac treatment in the presence of oseltamivir enabled selective imaging of cells transfected with oseltamivir-resistant NA gene with amino acid substitution of H275Y (Fig 3).

### Selective detection and isolation of oseltamivir-resistant viruses using live-focus fluorescence imaging

**Fig 4:**
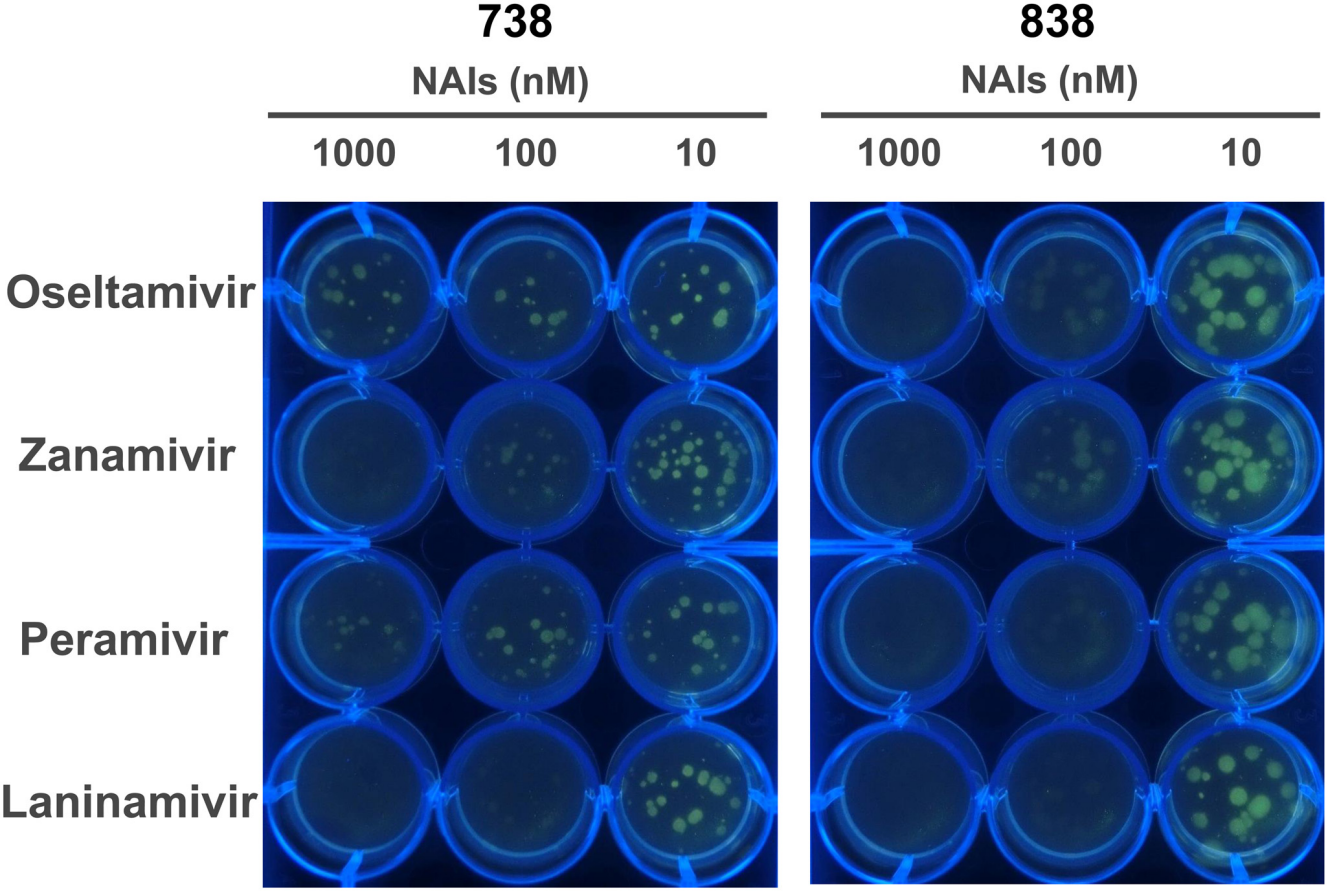
Live-focus fluorescence imaging of oseltamivir-resistant virus. MDCK cells on a 12-well plate were infected with oseltamivir-resistant 738 or oseltamivir-sensitive 838. The cells were cultured in an SFM containing 0.5% agarose and 2 μg/ml acetylated trypsin. After 2 days at 37°C, 50 μl of 2 mM BTP3-Neu5Ac with oseltamivir, zanamivir, peramivir or laninamivir (10, 100, 1000 nM for final concentrations) was added onto the overlaid agarose-containing SFM. After 1 hr at 37°C, fluorescent images of the plate were observed under UV irradiation at 365 nm.

We demonstrated that BTP3-Neu5Ac could detect NAI-resistant virus-infected cells. Some virus cultures and clinical samples are thought to contain a variety of NAI-resistant and -sensitive mutants, because of rich variations and liable mutations as a property of influenza virus. A mixture of NA-resistant and -sensitive viruses should show sialidase activity to some extent even in the presence of an NAI. In this case, it is necessary to determine whether a virus sample contains only an NAI-resistant virus or whether it contains various mutants and to efficiently isolate the NAI-resistant virus if NAI-resistant viruses were confirmed in the sample. We have developed live-focus fluorescence imaging that can fluorescent-visualize influenza A virus-forming focuses in a plaque-forming assay using BTP3-Neu5Ac, which is applicable to virus titration and virus isolation [26, 29]. The imaging is independent of various plaque-forming abilities among virus strains and can visualize as fluorescent focuses even virus strains that have no ability or little ability for plaque formation [29]. We predicted selective fluorescent visualization of NAI-resistant virus-forming focuses using BTP3-Neu5Ac with an NAI. MDCK cells were infected with 738 and 838 and cultured in an SFM containing 0.5% agarose and 2 μg/ml acetylated trypsin (required for infectious activation of progeny viruses) for 2 days. We tried selective fluorescent visualization of NAI-resistant virus-forming focuses by adding BTP3-Neu5Ac with clinical use-approved NAIs (oseltamivir, zanamivir, peramivir, and laninamivir) onto overlaid agarose-containing SFMs (Fig 4). Actually, focuses of oseltamivir-resistant 738 were distinctly detected using BTP3-Neu5A with 1000 nM oseltamivir (final concentration), but focuses of oseltamivir-sensitive 838 were not detected. There was no difference between 738 and 838 in sensitivities to zanamivir and laninamivir. Interestingly, oseltamivir-resistant 738 also showed distinct resistance against peramivir.

**Fig 5:**
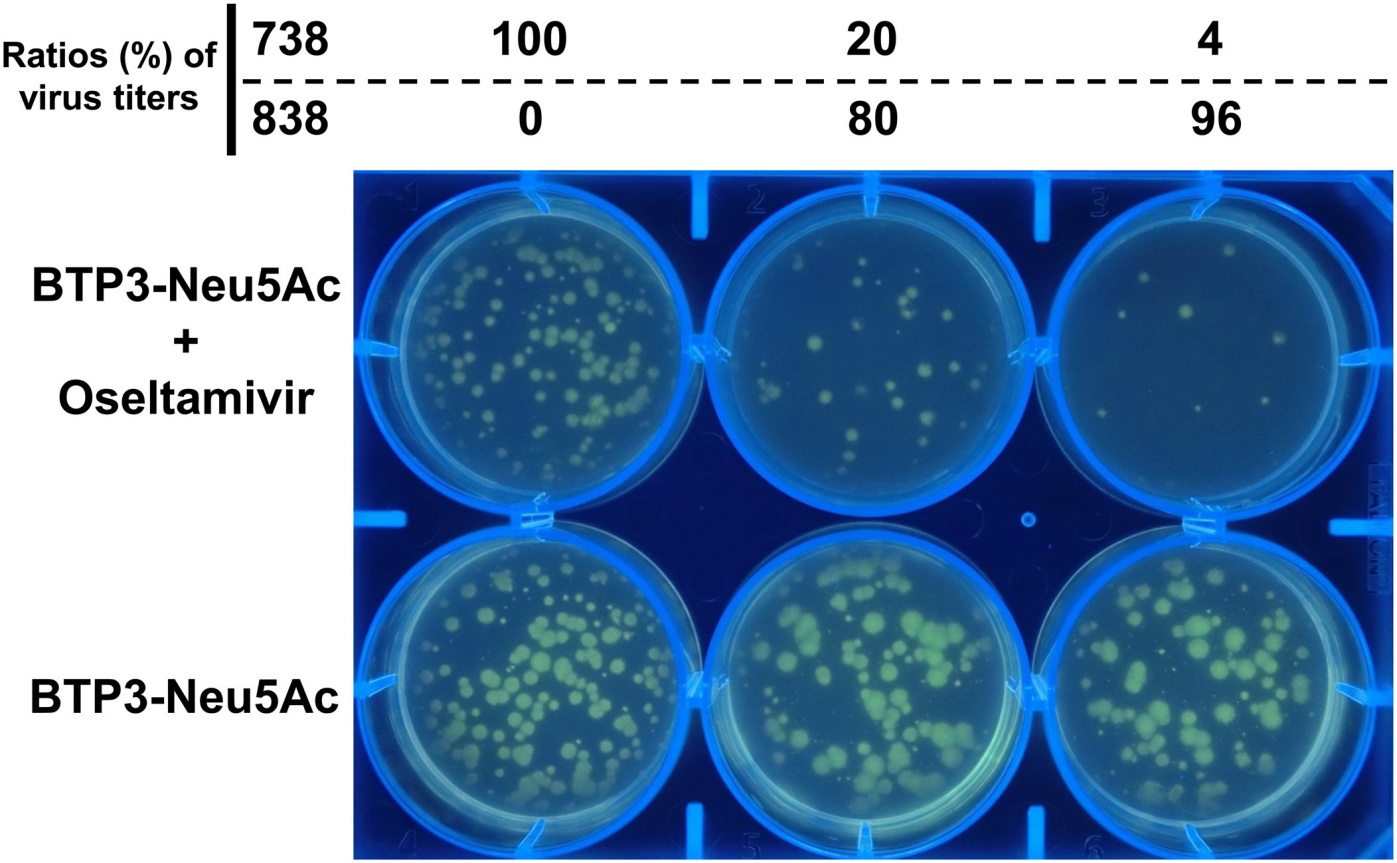
Selective live-focus fluorescence imaging of oseltamivir-resistant virus from a mixture of oseltamivir-sensitive and -resistant viruses. MDCK cells on a 6-well plate were infected with mixtures of oseltamivir-resistant 738 and -sensitive 838 at ratios (%) of 100:0, 20:80 and 4:96 in terms of virus titers (pfu). The infected cells were cultured in an SFM containing 0.5% agarose and 2 μg/ml acetylated trypsin. After 2 days at 37°C, 100 μl of 2 mM BTP3-Neu5Ac with or without oseltamivir (1000 nM for a final concentration) was dropped onto the overlaid agarose-containing SFM. After 1 hr at 37°C, fluorescent images of the plate were observed under UV irradiation at 365 nm.

In addition, we tried selective detection and isolation of an NAI-resistant virus from a mixture of NAI-resistant and -sensitive viruses. MDCK cells were infected with mixtures of oseltamivir-resistant 738 and -sensitive 838 at different ratios of virus titers. The cells were cultured in an SFM containing 0.8% agarose and 2 μg/ml acetylated trypsin for 2 days. All focuses were fluorescent-visualized by adding BTP3-Neu5Ac onto the overlaid agarose-containing SFM (Fig 5). The number of fluorescent focuses was similar at all ratios of virus mixtures, indicating that the infection titers were the same in all mixtures. On the other hand, when BTP3-Neu5Ac with 1000 nM oseltamivir (final concentration) was added onto the overlaid agarose-containing SFM, the number of fluorescent focuses increased according to the amount of oseltamivir-resistant 738 (Fig 5). Virus strains were isolated from fluorescent focuses in the presence of oseltamivir. To determine whether the isolates were 738 or 838, oseltamivir resistance mutation H275Y in NA (275Y for 738 and 275H for 838) was detected by RT-PCR from viral RNA. In three independent experiments of virus isolation, oseltamivir-resistant virus NA gene was detected for all of 24 isolates (S1 Fig). NAI-resistant viruses were obtained at a 100% success rate by live-focus fluorescence imaging using BTP3-Neu5Ac with an NAI. On the other hand, oseltamivir-sensitive virus NA gene was detected in 2 of 24 isolates, indicating contamination of oseltamivir-sensitive 838 to a selective isolate of oseltamivir-resistant 738. As a reason, it is thought that an invisible focus of oseltamivir-sensitive 838 overlapped with a fluorescent focus of oseltamivir-resistant 738.

## Discussion

We have developed a novel sialidase substrate, BTP3-Neu5Ac, for sensitive, rapid, and easy fluorescence imaging of cells infected with influenza A and B viruses. Influenza A virus has various antigenic subtypes (1–18 in HA and 1–11 in NA) [38] and there is the possibility of the occurrence of a pandemic as a new subtype of virus in humans [39]. Sialidase activity-based imaging by BTP3-Neu5Ac can be applied to almost all viral antigenicities, because the presence of sialidase activity is not dependent on any NA subtypes, except N10 and N11 subtypes showing no sialidase activity. In the present study, since an NAI-resistant virus retains sialidase activity even in the presence of NAI, we predicted that the use of BTP3-Neu5Ac with an NAI would enable selective fluorescent visualization of an NA-resistant virus and infected cells. We succeeded in selective detection and isolation of an NAI-resistant influenza virus from infected cells by live-cell (live-focus) fluorescence imaging using BTP3-Neu5Ac with an NAI. The present method will enable highly efficient detection and isolation of an NAI-resistant influenza virus even if the virus has undergone a drastic change on viral surface in a pandemic.

In surveillance and research of influenza virus sensitivity to NAIs, 4MU-Neu5Ac is most commonly used as a commercial sialidase substrate [40–43]. Since the product 4MU after sialidase reaction is a water-soluble fluorescent compound, 4MU-Neu5Ac cannot be used for live-cell fluorescence imaging. In the present study, we showed that sialidase activity-based imaging by BTP3-Neu5Ac can be used as a simple method for detecting cells infected with an NAI-resistant virus. In this method, the concentration of NAI is an important factor for clearly differentiating NAI-sensitive and -resistant viruses. IC_50_ values of oseltamivir in viral sialidase activity using 4MU-Neu5Ac were similar to those using BTP3-Neu5Ac. IC_50_ values reported in many previous studies using 4MU-Neu5Ac can be referred to for the determination of the appropriate concentration of NAI. Live-cell fluorescence imaging by BTP3-Neu5Ac with an NAI enables detection of an NAI-resistant virus at the cellular level, even very small titers of virus that cannot be detected by using 4MU-Neu5Ac. Moreover, after observation of fluorescent infected cells of an NAI-resistant virus, we can readily proceed to the virus culture.

Plaque-forming assay is a conventional virological method to measure infectious titers and to isolate a virus strain. Plaque-forming ability of a virus is generally dependent on the virus strain and cell type. Laboratory virus strains used in many previous studies have a tendency to form larger plaques. When such laboratory strains were used, plaques of an NAI-resistant virus are also selectively visualized by virus culture in an overlaid agarose-containing medium with an NAI, and the plaques have been usually used for isolation of an NAI-resistant virus [44, 45]. However, some virus strains including recent clinical strains have no ability or little ability for plaque formation [46, 47]. Live-focus fluorescence imaging by BTP3-Neu5Ac is a useful way to clearly visualize the focus of such a virus showing very small plaques or no plaques [29]. This great advantage of BTP3-Neu5Ac is also the same for focus visualization of an NAI-resistant virus in the presence of an NAI. Acquisition of NAI resistance mutations usually lowers the rate of virus growth compared to that of the NAI-sensitive parent virus, probably through reduction of sialidase activity [12–16]. Live focus imaging by BTP3-Neu5Ac will be more suitable than the conventional plaque-forming assay for the detection and isolation of NAI-resistant mutant viruses that show a lower rate of virus growth and form smaller plaques. In fact, for focus images at 10 nM NAIs shown in Fig 4, the plaque size of oseltamivir-resistant 738 appears to be smaller than that of oseltamivir-sensitive 838. However, live-focus fluorescence imaging by BTP3-Neu5Ac can clearly visualize a focus of both 738 and 838.

Isolation of NAI-resistant viruses is important for analysis of NAI resistance mutations. An NAI-resistant virus can be high-efficiently isolated from the fluorescent focus by live-focus fluorescence imaging using BTP3-Neu5Ac with an NAI. In the present study, selective isolation of an oseltamivir-resistant virus from mixtures of oseltamivir-resistant 738 and -sensitive 838 was also successfully performed by live-focus fluorescence imaging with oseltamivir. A representative method on identification of various NAI resistance mutations is to generate escape virus mutants through successive virus cultures in the presence of an NAI. Isolation of NAI-resistant escape mutants is essential to analyze mutations in the NA gene of each virus strain. For such study, live-focus fluorescence imaging by BTP3-Neu5Ac with an NAI is the most useful method to efficiently isolate NAI-resistant escape mutants selectively from virus cultures containing NAI-sensitive mutants and parent viruses. Counting the number of fluorescent focuses with and without NAI will also enable calculation of ratios of NAI-resistant and -sensitive viruses in clinical samples and virus cultures.

As shown in Fig 4, oseltamivir-resistant 738 was also resistant to peramivir. The chemical structure of peramivir possesses a guanidino group that appears in zanamivir and a hydrophobic pentyl group that appears in oseltamivir. H275Y mutation is associated with cross-resistance to oseltamivir and peramivir [48, 49]. At the stage of a public hygiene survey, 738 was confirmed to be an oseltamivir-resistant virus by genetic detection of H275Y mutation in NA but not a peramivir-resistant virus. Peramivir resistance of 738 was proved for the first time in the present study, although the peramivir resistance was predicted from the previous report about the cross-resistance between oseltamivir and peramivir. A method for genetic detection cannot be applied to unknown mutations conferring NAI resistance. A method for enzymatic detection, particularly a sensitive, rapid, and easy method, such as the use of BTP3-Neu5Ac, is suitable for cyclopaedically searching for resistant viruses against each clinical NAI.

Oseltamivir-resistant 738 was detected in all of 24 fluorescent focuses by live-focus fluorescence imaging with oseltamivir. On the other hand, oseltamivir-sensitive 838 was detected in 2 of 24 fluorescent focuses by the imaging, indicating contamination of 838 to 738 isolates. A probable reason is that an invisible focus of oseltamivir-sensitive 838 overlapped with a fluorescent focus of oseltamivir-resistant 738. In such a case, use of lower titers of a virus is supposed to reduce the risk of overlapping among focuses. The use of an agarose-containing medium with an NAI will reduce the focus size of an NAI-sensitive virus through inhibition of the growth of an NAI-sensitive virus. These methods would improve the efficiency of selective isolation of an NAI-resistant virus by live-focus fluorescence imaging.

We conclude that the use of BTP3-Neu5Ac with an NAI enables selective detection of an NAI-resistant virus at the levels of virus culture and infected cells (focus) in a sensitive, rapid, and easy manner. It is a powerful tool for public health workers who handle numerous samples and for researchers studying resistance mechanism of NAIs. It is also a useful tool for the development and screening of a novel NAI that has little risk of resistance acquisition and maintains effectiveness against NAs with various NAI-resistance mutations.

## Supporting Information

S1 Fig Selective isolation of oseltamivir-resistant virus using live-focus fluorescence imaging. (A) Viral RNA was extracted from virus cultures of oseltamivir-resistant 738 and -sensitive 838 strains. NA genes were amplified by RT-PCR from each viral RNA with UniNA-R primer and specific primers (08seasonNA-275Y-F and 09pdmNA-275H-F) for 275H or 275Y detection. (B) MDCK cells on a 6-well plate were infected with a mixture of oseltamivir-resistant 738 and -sensitive 838 strains at a ratio (%) of 1: 1 in terms of virus titers (pfu) (total of 40 pfu/well). The cells were cultured in an SFM containing 0.8% agarose and 2 μg/ml acetylated trypsin. After 2 days at 37°C, 100 μl of 2 mM BTP3-Neu5Ac with or without oseltamivir (at a final concentration of 1000 nM) was added onto the overlaid agarose-containing SFM. After 1 hr at 37°C, fluorescence images of the plate were observed under UV irradiation at 365 nm. Selective live-focus fluorescence imaging of oseltamivir-resistant 738 from the virus mixture was repeated three times by the same experimental method. Fluorescent images from each experiment are shown as “1st experiment”, “2nd experiment” and “3rd experiment”. Eight isolates per an experiment were obtained from fluorescent focuses using live-focus fluorescence imaging with oseltamivir. To distinguish genes of oseltamivir-resistant 738 and -sensitive 838 strains, 275H for 838 and 275Y for 738 in an NA amino acid difference were detected by RT-PCR from viral RNA of each isolate.
(DOC)
