# Supplementary material for: High-Efficiency Capture of Drug Resistant-Influenza Virus by Live Imaging of Sialidase Activity
Source: PLoS One. 2016 May 27;11(5):e0156400. doi: 10.1371/journal.pone.0156400 (PMC4883822; doi:10.1371/journal.pone.0156400)
Supplement: S1 Fig — (A) Viral RNA was extracted from virus cultures of oseltamivir-resistant 738 and -sensitive 838 strains. NA genes were amplified by RT-PCR from each viral RNA with UniNA-R primer and specific primers (08seasonNA-275Y-F and 09pdmNA-275H-F) for 275H or 275Y detection. (B) MDCK cells on a 6-well plate were infected with a mixture of oseltamivir-resistant 738 and -sensitive 838 strains at a ratio (%) of 1: 1 in terms of virus titers (pfu) (total of 40 pfu/well). The cells were cultured in an SFM containing 0.8% agarose and 2 μg/ml acetylated trypsin. After 2 days at 37°C, 100 μl of 2 mM BTP3-Neu5Ac with or without oseltamivir (at a final concentration of 1000 nM) was added onto the overlaid agarose-containing SFM. After 1 hr at 37°C, fluorescence images of the plate were observed under UV irradiation at 365 nm. Selective live-focus fluorescence imaging of oseltamivir-resistant 738 from the virus mixture was repeated three times by the same experimental method. Fluorescent images from each experiment are shown as “1st experiment”, “2nd experiment” and “3rd experiment”. Eight isolates per an experiment were obtained from fluorescent focuses using live-focus fluorescence imaging with oseltamivir. To distinguish genes of oseltamivir-resistant 738 and -sensitive 838 strains, 275H for 838 and 275Y for 738 in an NA amino acid difference were detected by RT-PCR from viral RNA of each isolate. (DOC) [file pone.0156400.s001.doc]

**Supporting Information**

**S1 Fig**

**S1 Fig. Selective isolation of oseltamivir-resistant virus using live-focus fluorescence imaging**
